# Shoe configuration effects on third phalanx and capsule motion of unaffected and laminitic equine hooves in-situ

**DOI:** 10.1371/journal.pone.0285475

**Published:** 2023-05-08

**Authors:** Rita Aoun, Iyana Charles, Abigail DeRouen, Catherine Takawira, Mandi J. Lopez

**Affiliations:** Laboratory for Equine and Comparative Orthopedic Research, Department of Veterinary Clinical Sciences, School of Veterinary Medicine, Louisiana State University, Baton Rouge, Louisiana, United States of America; China University of Mining and Technology, CHINA

## Abstract

Equine shoes provide hoof protection and support weakened or damaged hoof tissues. Two hypotheses were tested in this study: 1) motion of the third phalanx (P3) and hoof wall deformation are greater in laminitic versus unaffected hooves regardless of shoe type; 2) P3 displacement and hoof wall deformation are greatest while unshod (US), less with open-heel (OH), then egg-bar (EB) shoes, and least with heart-bar (HB) shoes for both hoof conditions. Distal forelimbs (8/condition) were subjected to compressive forces (1.0x10^2^–5.5x10^3^ N) while a real-time motion detection system recorded markers on P3 and the hoof wall coronary band, vertical midpoint, and solar margin. Magnitude and direction of P3 displacement and changes in proximal and distal hemi-circumference, quarter and heel height and proximal and distal heel width were quantified. Hoof condition and shoe effects were assessed with 2-way ANOVA (p<0.05). P3 displacement was greater in laminitic hooves when US or with OH, and EB and HB reduced P3 displacement in laminitic hooves. P3 displacement was similar among shoes in unaffected hooves and greatest in laminitic hooves with OH, then US, EB and HB. EB and HB increased P3 displacement from the dorsal wall in unaffected hooves and decreased it in laminitic hooves. OH and EB increased P3 motion from the coronary band in laminitic hooves, and HB decreased P3 motion toward the solar margin in unaffected and laminitic hooves. In laminitic hooves, HB reduced distal hemi-circumference and quarter deformation and increased heel deformation and expansion. Proximal hemi-circumference constriction was inversely related to proximal heel expansion with and without shoes. Overall, shoe configuration alters hoof deformation distinctly between unaffected and laminitic hooves, and HB provided the greatest P3 stability in laminitic hooves. These unique results about P3 motion and hoof deformation in laminitic and unaffected hooves inform shoe selection and design.

## Introduction

Equine laminitis is a multi-factorial condition that occurs in 2.4% to 33.8% of horses with a high incidence of recurrence and/or transition to a chronic state [1–4]. The condition is related to endocrinopathy (metabolic) [5–7], systemic inflammatory response syndrome [8, 9], and excessive weightbearing on a supporting limb [10, 11] among other inciting pathologies. Regardless of etiology, there are established, microscopic changes in the hoof tissue that can progress to separation of the secondary epidermal and dermal lamellae that connect the outer hoof shell to the third phalanx (P3) [2, 12]. Subsequently, P3 may rotate or sink within the hoof capsule, and poorly organized fibrous tissue often replaces the intricate architecture of the epidermal-dermal interface as a lamellar wedge of scar horn [2, 7, 13, 14]. Irreversible hoof changes can result in intractable pain and altered structural function. The higher incidence and greater severity of laminitis in the forelimbs is thought to be related, in part, to the higher percentage of body weight borne relative to the hind limbs, 68%:42% [15–18].

Treatment of laminitis often includes a broad spectrum of therapies to address the underlying cause of the condition, manage pain, and maintain hoof circulation [19, 20]. A cornerstone of therapeutic intervention includes protection of the weakened hoof tissue with deep, soft bedding and coaptive hoof support to reduce the forces on the soft tissue hoof interface and help prevent P3 displacement and rotation [21]. While vertical forces clearly contribute to vertical displacement or sinking of P3, tensile forces from the deep digital flexor tendon (DDFT), are thought to contribute to rotation or angular displacement of the dorsal aspect of P3 away from the hoof wall [22, 23]. Supportive shoeing is typically designed to counteract the forces by distributing load over a greater surface area to reduce that on the toe and/or by effecting heel elevation to lower the tensile DDFT forces on P3 [21, 24, 25].

Egg-bar and heart-bar are examples of shoes designed to increase the hoof contact surface area to include the palmar/plantar hoof and displace the center of force in the caudal direction to reduce weight bearing on the toe [1, 26–29]. The heart-bar shoe increases the load distribution over that of the egg-bar by inclusion of the frog surface area [30–32]. Ground reaction forces, contact surface area and pressure, wall strains, and circulation have been investigated for both shoe configurations [33–36]. Quantification of shoe configuration effects on P3 motion is vital for preventing rotation and/or sinking of the structure [23, 37–39], both of which are associated with a poor prognosis [11, 40–42]. To date, however, there is comparably little information on the direct impact of the shoes on P3 motion, especially relative to hoof wall surfaces. Quantification of the changes in hoof wall deformation and P3 motion conferred by shoe configuration is important for the design and application of equine shoes, especially those designed to stabilize or immobilize hoof structures during weight bearing and locomotion.

**Fig 1:**
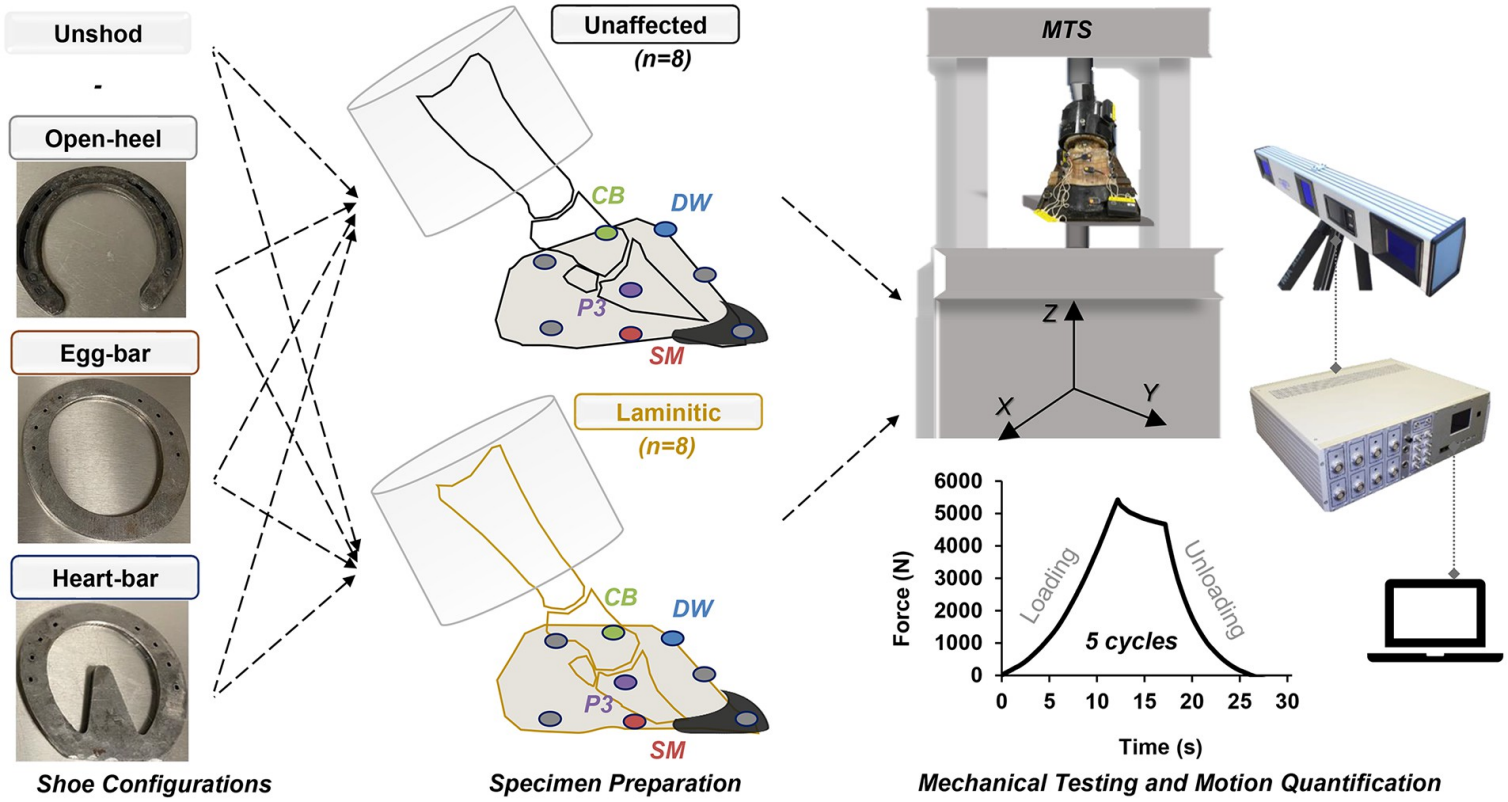
Study design schematic. Unaffected and laminitic hooves (n = 8/condition) were stabilized in fixtures on a materials testing system at the metacarpophalangeal joint proximally and the toe distally. Infrared markers of an active motion detection system were attached to the third phalanx (P3) through a window in the hoof capsule and the hoof wall at the level of the coronary band and the solar margin of the lateral and medial quarters and heels. Additional markers were placed at the vertical midpoint of the hoof wall at the toe and the medial quarter, at the dorsal toe at the level of the coronary band, and on the testing fixture at point of the toe. Motion data was recorded during loading up to 5.5x10^3^ N in a pattern representing a forelimb step cycle with hooves unshod and with open-heel, egg-bar, and heart-bar shoes applied in random order. DW: dorsal wall coronary band, CB: lateral quarter coronary band, SM: lateral quarter solar margin.

The purpose of this in-situ study was to investigate the stabilizing effects of open-heel, egg-bar and heart-bar shoes on P3 and the adjacent hoof components during application of vertical stance forces of a walking step cycle (Fig 1). The two hypotheses tested were: 1) motion of P3 and hoof wall deformation are greater in laminitic versus unaffected hooves regardless of shoe type; and 2) P3 displacement and hoof wall deformation are greatest while unshod, less with open-heel, then egg-bar shoes, and least with heart-bar shoes applied in laminitic and unaffected hooves. Quantification of changes in hoof wall and P3 motion affected by shoe configuration will contribute to design and application of shoes to stabilize and protect sensitive hoof structures, especially those weakened by laminitis.

## Materials and methods

### Specimen collection and preparation

With the owner’s consent, equine digits were harvested from forelimbs of horses euthanized for reasons unrelated to the study. Hooves without (unaffected) and with (laminitic) chronic laminitis (n = 8/condition) were disarticulated at the metacarpophalangeal joint immediately post-mortem, wrapped in saline-soaked towels, then sealed in plastic bags, and stored at -20ºC. Age, weight, sex, breed, and medical history were recorded for each specimen. Specimen inclusion criteria were: 1) 3–15 years; 2) 400–550 kg; 3) light breed; 4) mare or gelding; 5) intact hoof capsule (no detectable abscesses or sole or wall disruption); 6) no forelimb lameness for the unaffected condition or Obel grade 1–2 for at least 6 weeks for the laminitic condition.

**Fig 2:**
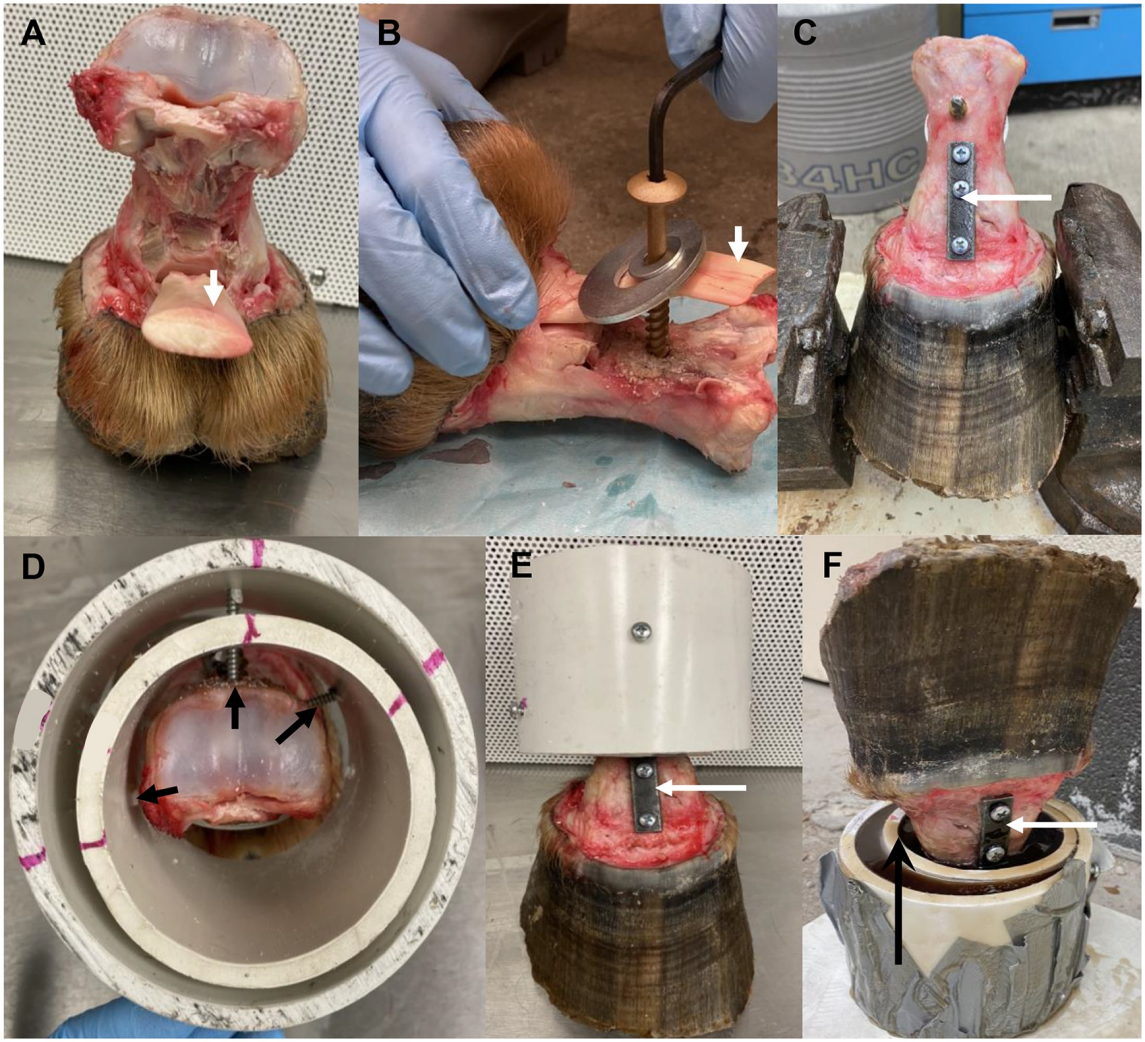
Specimen preparation steps. Photographs depicting a forelimb digit (A) with soft tissues removed except the deep digital flexor tendon (DDFT, white arrow, A, B); fixation of the DDFT to the first phalanx (B); a 3-hole plate (white arrow, C, E, F) immobilizing the proximal interphalangeal joint (C); a specimen stabilized with screws (black arrows, D) within two PVC pipes viewed from proximal (D) and dorsal (E) perspectives; and a construct with resin surrounding the specimen and between two PVC pipes (black arrow, F).

To prepare for testing, hooves were thawed in saline at room temperature overnight (20°C), and all soft tissues proximal to the coronary band, except the DDFT, were removed with care not to disrupt the distal interphalangeal joint capsule and intracapsular hoof tissues (Fig 2). A pilot hole (5.5 mm) was drilled in the caudal cortex of the first phalanx approximately 3 cm from the proximal articular surface at the transverse midpoint, and the DDFT was affixed to the bone with a 8 mm x 10 cm (diameter x length) wood screw (Hillman Group, Inc, Cincinnati, OH, USA) and two, stacked, flat stainless steel washers with 1 cm and 4 cm diameters (Hillman Group, Inc, Cincinnati, OH, USA), with the largest washer closest to the tendon. The proximal interphalangeal joint was immobilized with a dorsal 3-hole, stainless steel plate (50 x 12.5 x 3 mm, length, width, height) and 5.5 mm x 5 cm cortical screws (OrthoMed, Inc, Huddersfield, UK) using standard Association for the Study of Internal Fixation technique after aligning the dorsal surface of the proximal and middle phalanges. The proximal and distal plate holes were 1.5 cm and 2.5 cm away from the middle hole, respectively, and the distal edge of the plate was approximately 2.5 cm proximal to the coronary band.

The first phalanx and the proximal half of the second phalanx were stabilized within a polyvinyl chloride (PVC) pipe (7.5 x 10 cm) within another PVC pipe (10 x 10 cm) with three cabinet mounting screws (4.8 mm x 7.6 cm) placed through holes in the pipes on the dorsal, medial, and lateral surfaces. Duct tape was used to seal the most proximal surface of the construct, which was subsequently inverted, and the spaces between the specimen and pipe and between the pipes filled with fiberglass polyester resin (Bondo^®^, 3M, Atlanta, Georgia, USA). The specimens were allowed to solidify overnight at room temperature (20°C). Subsequently, a 2.5 cm diameter tunnel was created through the lateral hoof capsule to P3 with a 1.9 cm-diameter circular hole saw (Morse Cutting Tools, Novi, Michigan, USA) on a low-speed drill at the point where lines between the greatest height and greatest width of the lateral hoof surface intersected. The tunnel was cleared of debris and the exposed surface of P3 gently debrided with a #20 scalpel blade. A 3 mm-diameter drill bit and low-speed drill was used to create a 1 cm deep tunnel in P3 into which a 3.5 mm x 3.6 cm cortical self-tapping screw (OrthoMed, Inc, Huddersfield, UK) was inserted such that the top of the screw head was level with the hoof wall surface. Specimens were stored at -20ºC and then thawed as described above prior to mechanical testing.

### Mechanical testing and hoof motion capture

A certified farrier trimmed and leveled all hooves prior to shoe application and applied correctly sized shoes throughout the study. Shoes, including open-heel, egg-bar, and heart-bar, were affixed with four nails (two per side) to avoid multiple nail holes in the hoof wall. The proximal aspect of each specimen was secured in a 14 cm diameter x 9.5 cm deep receiving cup attached to a 100 kN (axial) load cell (Densor Developments Inc., Orion, Michigan, USA) of a materials testing system (809 Axial/Torsional Testing System, Manufacturing Technical Solutions, Inc., Eden Prairie, MN, USA) by means of a stainless steel rod (3 cm x 20 cm) at a 140° angle to the palmar aspect of the hoof. The hoof rested on a stainless-steel plate (30 x 20 x 1 cm) attached to the actuator with an adjustable, stainless steel, curved toe piece that lightly approximated the distal toe surface to prevent the specimen from sliding forward without constraining hoof wall expansion.

**Fig 3:**
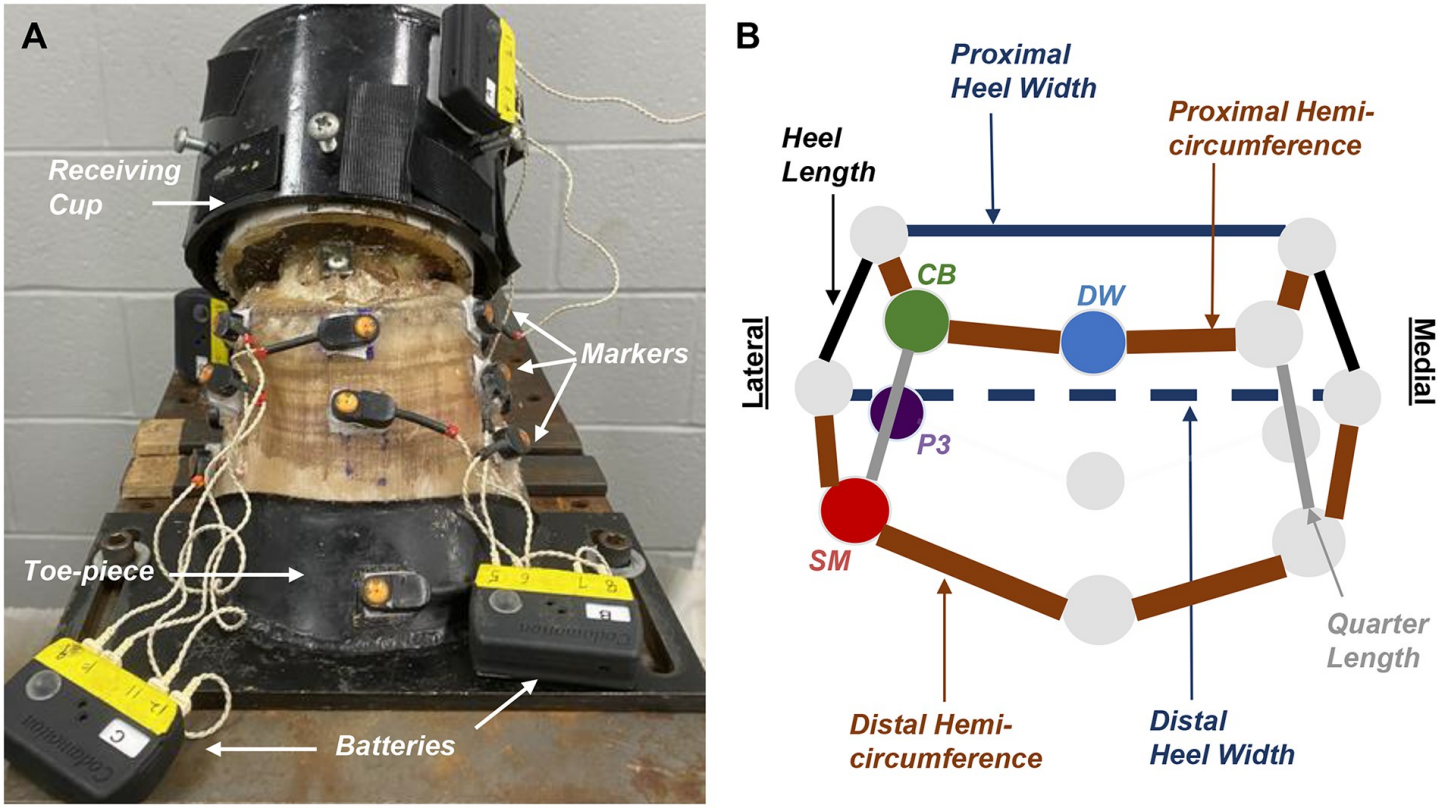
Equine hoof instrumented for data acquisition. Representative specimen with infrared markers attached and stabilized between the receiving cup (proximally) and the toe piece (distally) fixtures (A) and a corresponding stick figure diagram showing individual segments between markers used to quantify proximal and distal hemi-circumferences, heel and quarter lengths, and proximal and distal heel widths (B). The spheres in the stick figure diagram correspond to the infrared markers, and the lateral surface of the hoof is to the left in each image. DW: dorsal wall coronary band, CB: lateral quarter coronary band, SM: lateral quarter solar margin.

Light emitting diode markers (LED, 2 x 1 cm) of an active motion detection system (Codamotion, Charnwood Dynamics, Ltd, Rothley, UK) were attached to the hoof wall at the level of the coronary band and the solar margin of the lateral and medial quarters and heels (Fig 3). Markers were also placed at the vertical midpoint of the hoof wall at the toe and the medial quarter, and a marker was attached to the head of the screw in the lateral surface of P3. An additional marker was placed on the toe piece fixture at the point of the toe solar margin. The markers were attached using commercially available hook and loop fasters (1 x 1 cm, Velcro^®^, Velcro USA, Inc, Manchester, NH, USA) adhered to the markers and hoof surfaces with cyanoacrylate glue. This allowed the markers to be removed and reapplied in identical positions when shoes were replaced between trials. Two optoelectronic sensor units (Codamotion) were placed 3 m from the center of the testing fixtures with one facing the lateral and the other facing the medial hoof surface. A fixed origin of a virtual cartesian system coordinate axis was established at the left bottom corner of the testing machine for all trials. After marker application, specimens were preloaded to 100 N and then loaded at a rate of 1 mm/sec (~350 N/s) to 5.5x10^3^ N where the displacement was held for 5 seconds followed by unloading to 100 N at the same rate for 5 cycles. Simultaneous load and motion data were recorded at a rate of 100 Hz and marker location at a rate of 200 Hz (ODIN, Codamotion).

### Data reduction

The three-dimensional (3-D) displacement of the lateral and dorsal hoof wall landmarks and P3 were determined from a total of 4 markers, the dorsal wall (DW) coronary band, lateral quarter coronary band (CB) and solar margin (SM), and P3 (Fig 1). Cartesian coordinates at 5% load increments over the loading cycle were used to calculate the 3-D displacement of each marker relative to the position at the initial 100 N load as $D=\sqrt{{\left(x_{l}-x_{0}\right)}^{2}+{\left(y_{l}-y_{0}\right)}^{2}+{\left(z_{l}-z_{0}\right)}^{2}}$ where D is the displacement in mm, *x*_*l*_, *y*_*l*_, and *z*_*l*_ are the marker coordinates at each load interval, and *x*_0_, *y*_0_, and *z*_0_ are the marker coordinates at 100 N (x axis = 0). The Cartesian coordinates of the same markers at 20% intervals were also used to quantify the 3-D displacement of P3 relative to points DW, CB and SM using the formula $\Delta D=\sqrt{{\left(x_{P3l}-x_{ml}\right)}^{2}+{\left(y_{P3l}-y_{ml}\right)}^{2}+{\left(z_{P3l}-z_{ml}\right)}^{2}}-\sqrt{{\left(x_{P30}-x_{m0}\right)}^{2}+{\left(y_{P30}-y_{m0}\right)}^{2}+{\left(z_{P30}-z_{m0}\right)}^{2}}$ where ΔD is the change in distance between P3 and each distinct landmark, *x*_*P*3*l*_, *y*_*P*3*l*_, and *z*_*P*3*l*_ are the P3 coordinates and *x*_*ml*_, *y*_*ml*_, and *z*_*ml*_ are DW, CB, or SM coordinates at a given load interval while *x*_*P*30_, *y*_*P*30_, *z*_*P*30_ and *x*_*r*0_, *y*_*r*0_, *z*_*r*0_ are the P3 and DW, CB or SM coordinates at 100 N. An increase in distance between two markers on the hoof at a given loading stage relative to the distance at 100 N is indicated by a positive numeric result (points are moving away from each other) while a decrease in distance is indicated by a negative numeric result (points are moving toward each other).

The combined 3-D distance between markers from the medial heel through the medial quarter, toe, and lateral quarter to the lateral heel at the level of the coronary band, the proximal hoof hemi-circumference, and at the level of the solar margin, the distal hoof hemi-circumference, was determined at 20% intervals over the loading cycle. The displacement of each hemi-circumference was quantified using the formula $\Delta L=\sum_{i=1}^{4}\sqrt{{\left(x_{\left(i+1\right)l}-x_{\left(i\right)l}\right)}^{2}+{\left(y_{\left(i+1\right)l}-y_{\left(i\right)l}\right)}^{2}+{\left(z_{\left(i+1\right)l}-z_{\left(i\right)l}\right)}^{2}}-\sum_{i=1}^{4}\sqrt{{\left(x_{\left(i+1\right)0}-x_{\left(i\right)0}\right)}^{2}+{\left(y_{\left(i+1\right)0}-y_{\left(i\right)0}\right)}^{2}+{\left(z_{\left(i+1\right)0}-z_{\left(i\right)0}\right)}^{2}}$, where Δ*L* is the sum of the 3-D distance between the markers along the proximal or distal hemi-circumference normalized to the total length at 100 N; *x*_(*i*)*l*_, *y*_(*i*)*l*_, *z*_(*i*)*l*_ and *x*_(*i*+1)*l*_, *y*_(*i*+1)*l*_, *z*_(*i*+1)*l*_ are the Cartesian coordinates of each marker and its adjacent marker, in a clockwise direction, at a given load *l*, and *x*_(*i*)0_, *y*_(*i*)0_, *z*_(*i*)0_ and *x*_(*i*+1)0_, *y*_(*i*+1)0_, *z*_(*i*+1)0_ are the marker coordinates at 100 N.

The change in 3-D distance between markers on the coronary band and solar margin of each quarter and heel was also quantified at 20% load intervals using the formula $\Delta L=\sqrt{{\left(x_{(i+1)l}-x_{(i)l}\right)}^{2}+{\left(y_{(i+1)l}-y_{(i)l}\right)}^{2}+{\left(z_{(i+1)l}-z_{(i)l}\right)}^{2}}\;-\;\sqrt{{\left(x_{(i+1)0}-x_{(i)0}\right)}^{2}+{\left(y_{(i+1)0}-y_{(i)0}\right)}^{2}+{\left(z_{(i+1)0}-z_{(i)0}\right)}^{2}}$, where Δ*L* is the change in distance, or 3-D length, between the two markers on the quarters or heels at each load increment normalized to the initial length at 100 N (baseline); *x*_(*i*)*l*_, *y*_(*i*)*l*_, *z*_(*i*)*l*_ and *x*_(*i*+1)*l*_, *y*_(*i*+1)*l*_, *z*_(*i*+1)*l*_ are the marker Cartesian coordinates at a given load *l* and *x*_(*i*)0_, *y*_(*i*)0_, *z*_(*i*)0_ and *x*_(*i*+1)0_, *y*_(*i*+1)0_, *z*_(*i*+1)0_ are the marker coordinates at 100 N. The mean of the medial and lateral quarter or heel change in 3-D length at each load increment was used as the data point for purposes of statistical analysis.

Similarly, the change in 3-D distances between medial and lateral heel markers on the proximal or distal heel, the proximal and distal heel width, respectively, were quantified at 20% load intervals using the formula $\Delta L=\sqrt{{\left(x_{(i+1)l}-x_{(i)l}\right)}^{2}+{\left(y_{(i+1)l}-y_{(i)l}\right)}^{2}+{\left(z_{(i+1)l}-z_{(i)l}\right)}^{2}}\;-\;\sqrt{{\left(x_{(i+1)0}-x_{(i)0}\right)}^{2}+{\left(y_{(i+1)0}-y_{(i)0}\right)}^{2}+{\left(z_{(i+1)0}-z_{(i)0}\right)}^{2}}$, where Δ*L* is the change in distance between the proximal or distal, lateral and medial markers on the heel at each load increment normalized to the initial length at 100 N (baseline); *x*_(*i*)*l*_, *y*_(*i*)*l*_, *z*_(*i*)*l*_ and *x*_(*i*+1)*l*_, *y*_(*i*+1)*l*_, *z*_(*i*+1)*l*_ are the marker Cartesian coordinates at a given load *l* and *x*_(*i*)0_, *y*_(*i*)0_, *z*_(*i*)0_, and *x*_(*i*+1)0_, *y*_(*i*+1)0_, *z*_(*i*+1)0_ are the marker coordinates at baseline.

### Radiographic hoof imaging during loading

**Fig 4:**
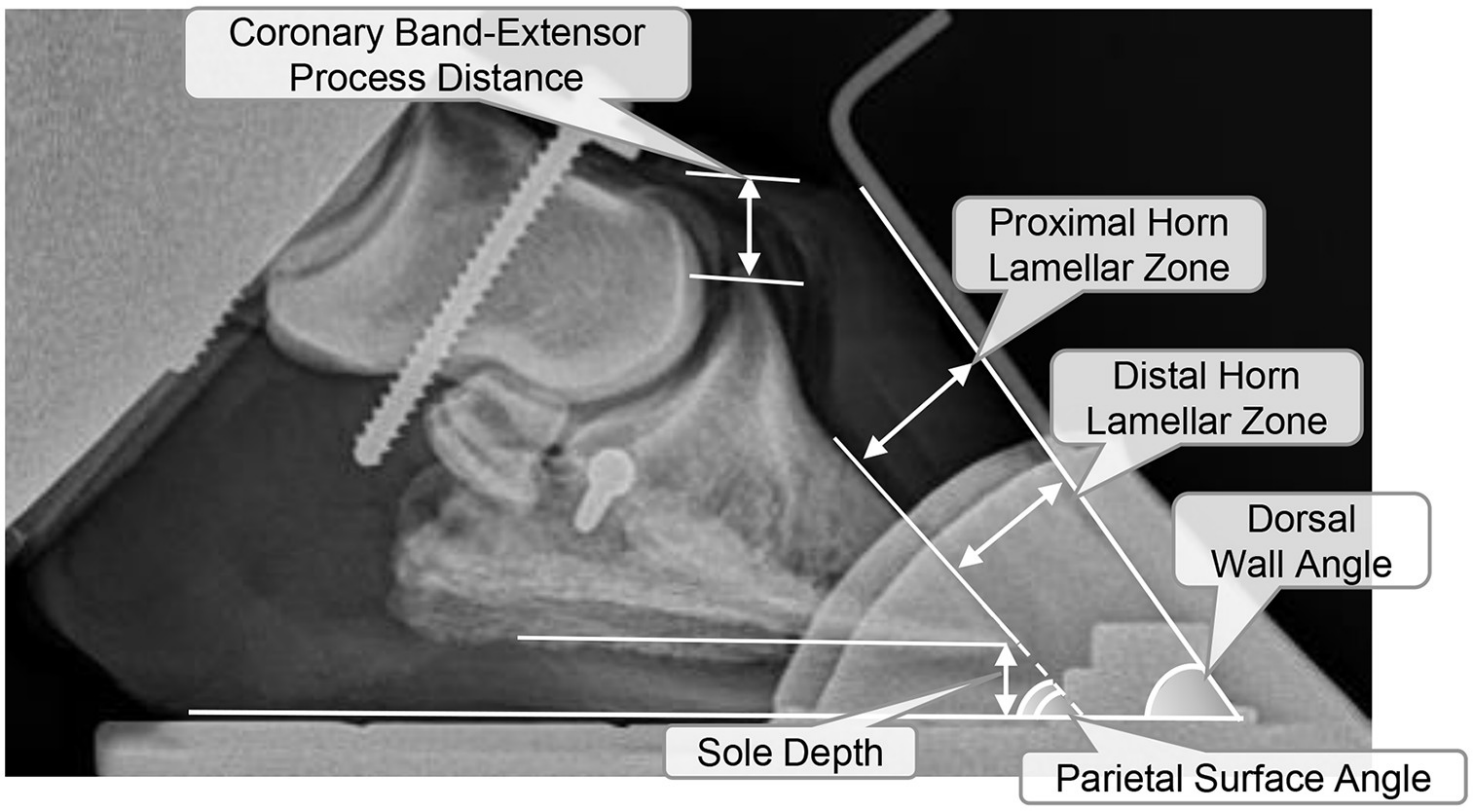
Measures on a mediolateral equine hoof radiograph. Representations of the radiographic measures used in the study including the coronary band to P3 extensor process distance (CE), proximal and distal horn lamellar zone (HL), the dorsal wall and P3 parietal surface angles, and sole depth (S).

Mediolateral radiographs of each unshod hoof were obtained with a portable digital radiography system (68 kV, 0.08 mA/sec, MinXray HF8015+, MinXray, Inc., Northbrook, Illinois, USA) with constructs in the testing fixtures described above and during static loading at 100, 1375, 2750, 4125, and 5500 N, corresponding to preloading and 25, 50, 75 and 100% of maximum dynamic loading described above. The long arm of a 7 mm-diameter Allen wrench (5.1 x 1.6 cm) was secured to the dorsal hoof wall and used as a boundary for standard measures on radiographs generated at each loading interval [43]. Measures included: distance from the coronary band to the P3 extensor process (CE); distance from the dorsal hoof wall to the proximal and distal third of the dorsal P3 surface, the horn lamellae (HL) zone; the distance from the ground surface to the tip of P3, the sole depth (S); and the dorsal wall and P3 parietal surface angles, the angle between the dorsal hoof wall surface and the ground, and the angle between the P3 parietal surface and the ground, respectively (Fig 4).

### Statistical analysis

A preliminary power analysis at 80% power and an effect size of 0.35 using means of P3 displacement in laminitic and unaffected hooves from preliminary studies gave a total sample size of 8 per cohort for a MANOVA with a design of two groups and four repeated measurements (G*Power, University of Düsseldorf, Düsseldorf, Germany). Results are reported as mean ± SEM. All outcome measures were tested for normality with the Shapiro-Wilk test. A two-way analysis of variance (ANOVA) was performed to test the effect of load and condition (unaffected or laminitic) on individual marker displacement and the effect of load and shoe configurations on displacement change. When main effects were determined, Tukey’s post-hoc tests were applied to determine differences among conditions or shoe treatments. Statistical significance was considered at p ≤ 0.05.

## Results

### Lateral and dorsal hoof wall and P3 3-D displacement

**Fig 5:**
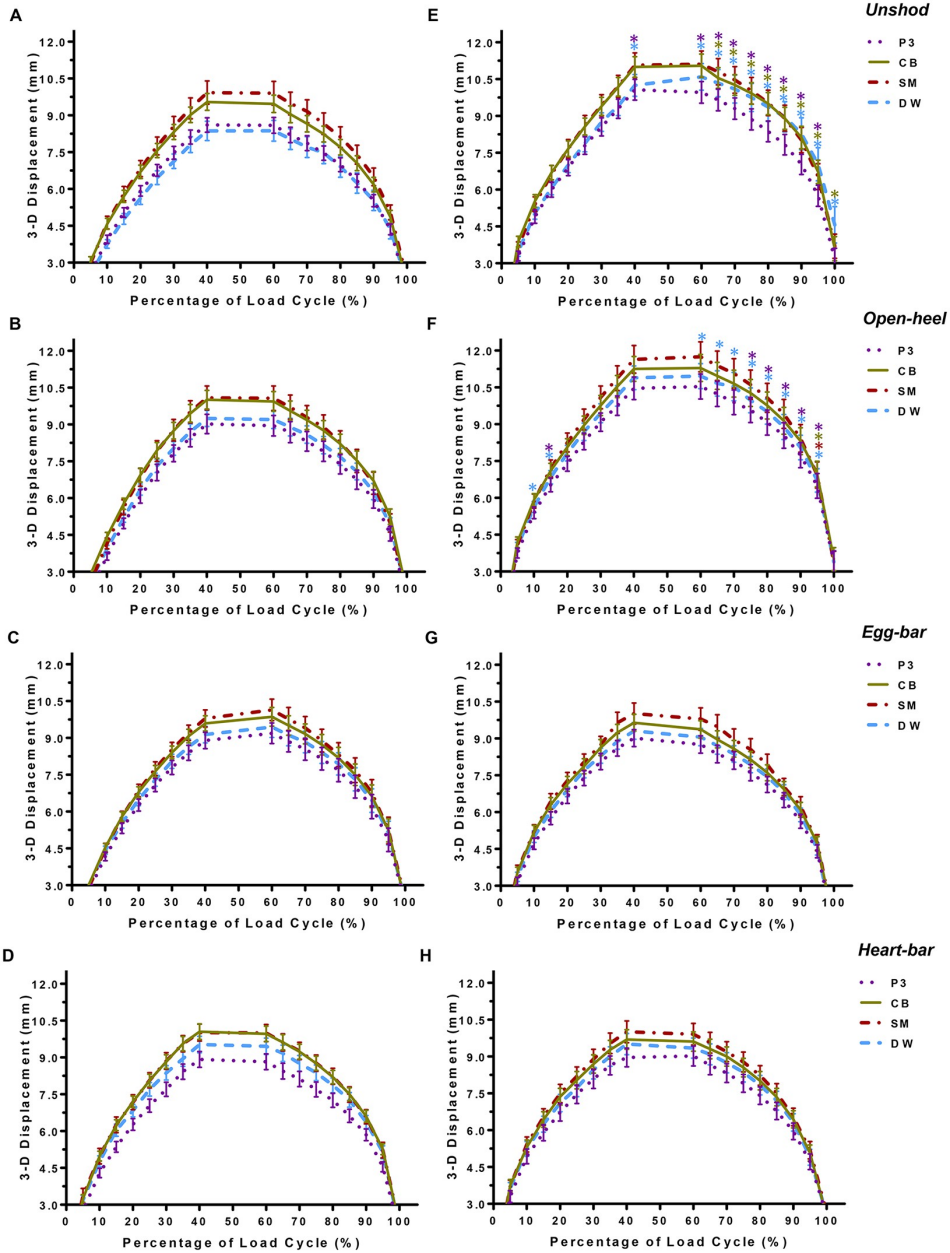
Three-dimensional hoof landmark displacement. Displacement curves (mean ± SEM) of the third phalanx (P3), lateral quarter coronary band (CB), lateral quarter solar margin (SM), and dorsal wall coronary band (DW) of unaffected (A-D) and laminitic (E-H) hooves during application of a compressive load cycle (~350N/s) from 1.0x10^2^*–*5.5x10^3^ N (n = 8/condition) while unshod (A, E) or while shod with open-heel (B, F), egg-bar (C, G), or heart-bar (D, H) shoes. Significant differences in hoof marker displacement between unaffected and laminitic hooves for each shoeing condition are indicated by asterisks above the associated points in the load cycle (p ≤ 0.05). Asterisk color corresponds to marker location.

The 3-D displacement of lateral hoof wall landmarks increased with increasing load in both unaffected and laminitic hooves (Fig 5). The displacement of the SM was generally greatest, followed by the CB, and it was lowest in DW and P3 in both unaffected and laminitic hooves for all shoeing conditions. Markedly, DW displacement was lower than P3 in unaffected hooves while unshod, but higher than P3 with all shoes tested.

The shape of the displacement curves for all markers over the course of loading and unloading of unaffected hooves had a fairly uniform semi-circular shape with and without shoes while those of laminitic hooves were taller and more elliptical while unshod and shod with open-heel shoes. In unshod hooves, the P3 and DW, and P3, CB, and DW displacement was higher in laminitic versus unaffected hooves at the highest load, and during unloading, respectively. Additionally, while shod with open-heel shoes, the DW and P3 displacement in laminitic hooves was greater than unaffected hooves at the lowest load, DW displacement at the highest load, P3 and DW during unloading, and for P3, CB, DW, and SM at the end of unloading. Following application of egg-bar and heart-bar shoes, there were no differences in 3-D displacement between laminitic and unaffected hooves at any hoof landmark, and the displacement curves had similar shapes.

**Fig 6:**
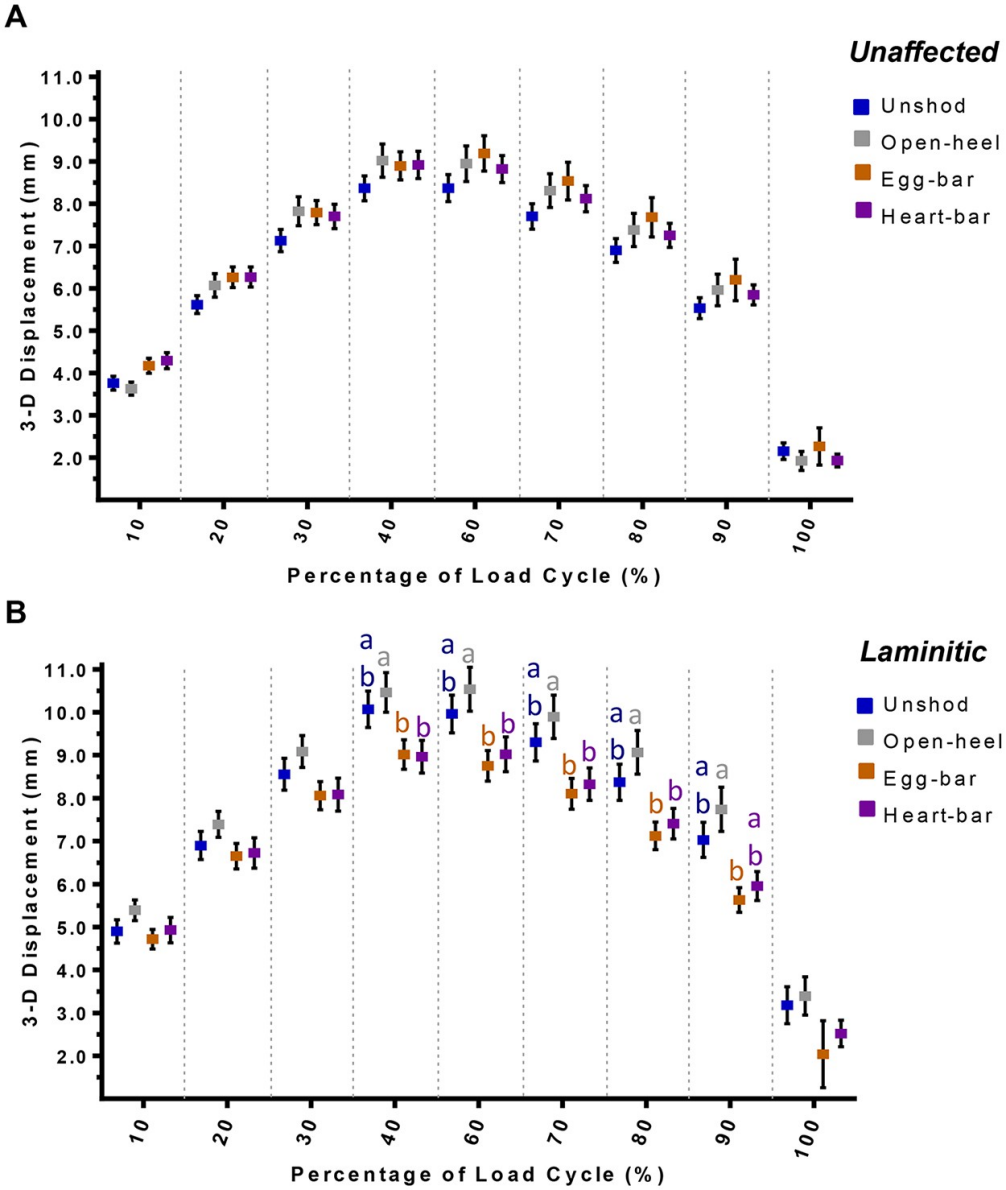
Three-dimensional P3 displacement. Three-dimensional P3 displacement (mean ± SEM) in unaffected (A) and laminitic (B) equine hooves during application of a compressive load cycle (~350N/s) from 1.0x10^2^–5.5x10^3^ N (n = 8/condition) while unshod (blue) or while shod with open-heel (gray), egg-bar (orange), or heart-bar (purple) shoes. Significant differences among shoes within hoof condition at distinct loading cycle percentages are indicated by different color letters above data points (p≤0.05).

Overall, P3 displacement in laminitic hooves was lower with egg-bar and heart-bar shoes compared to unshod and with open-heel shoes (Fig 6). Specifically, P3 displacement was significantly lower with egg-bar and heart-bar shoes at the maximum force and during the unloading phase of the cycle compared to open-heel.

### 3-D P3 displacement relative to DW, CB and SM

**Fig 7:**
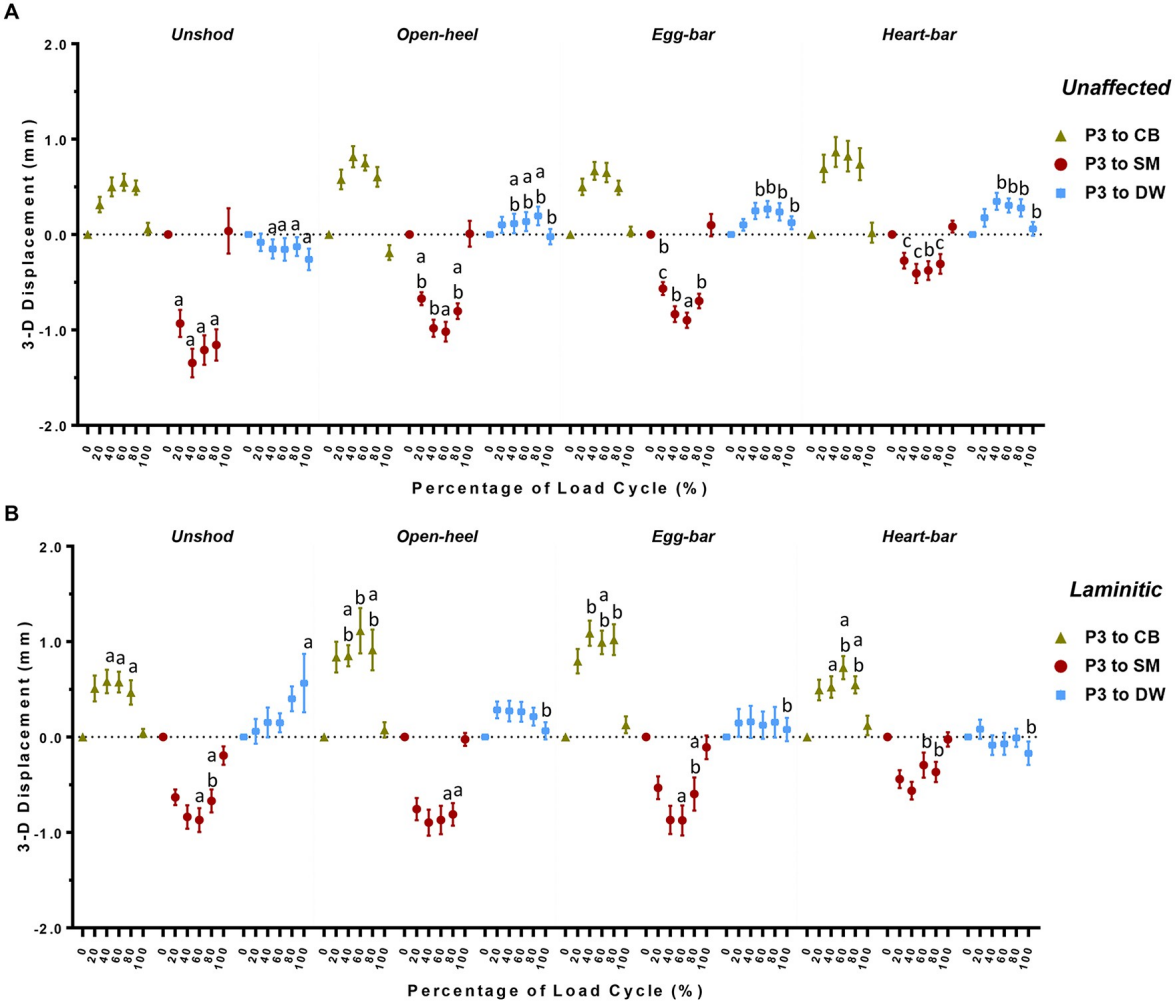
P3 displacement direction. Three-dimensional displacement (mean ± SEM) of P3 relative to the lateral quarter coronary band (CB), lateral solar margin (SM) and dorsal wall coronary band (DW) in unaffected (A) and laminitic (B) equine hooves during application of a compressive load cycle (~350N/s) from 1.0x10^2^–5.5x10^3^ N (n = 8/condition) while unshod or while shod with open-heel, egg-bar, or heart-bar shoes. Significant differences in landmark displacements among shoeing conditions at the indicated percentage of the load cycle are indicated by distinct letters above data points (p≤0.05).

Significant differences in 3-D P3 displacement relative to distinct hoof landmarks in unaffected hooves included significantly higher displacement away from DW at the highest loading states and during unloading while shod with egg-bar and heart-bar shoes compared to unshod (Fig 7). Additionally, P3 displacement toward SM was significantly greater while unshod than when shod with heart-bar shoes and tended to be higher than with egg-bar shoes at all loading stages. The 3-D distance between P3 and SM was similar in unaffected hooves shod with open-heel or egg-bar shoes, and both were less than with heart-bar shoes during most of the loading cycle.

The displacement between hoof landmarks and P3 in laminitic hooves was distinct from unaffected hooves in that significant differences among shoeing conditions were primarily in displacement away from CB, however, at the last point of the unloading phase, the displacement of P3 away from DW was greatest in unshod compared to all shoeing conditions. During the unloading phase, P3 displacement toward SM was significantly lower when hooves were shod with heart-bar shoes versus open-heel shoes, and the displacement with open-heel shoes was similar to that with egg-bar shoes or while unshod. The P3 displacement away from CB was greater with egg-bar shoes during the loading and unloading stages except at the second point of highest load and with open-heel shoes only at the initial highest loading point compared to unshod.

### Hoof wall hemi-circumference, quarter, and heel length changes

**Fig 8:**
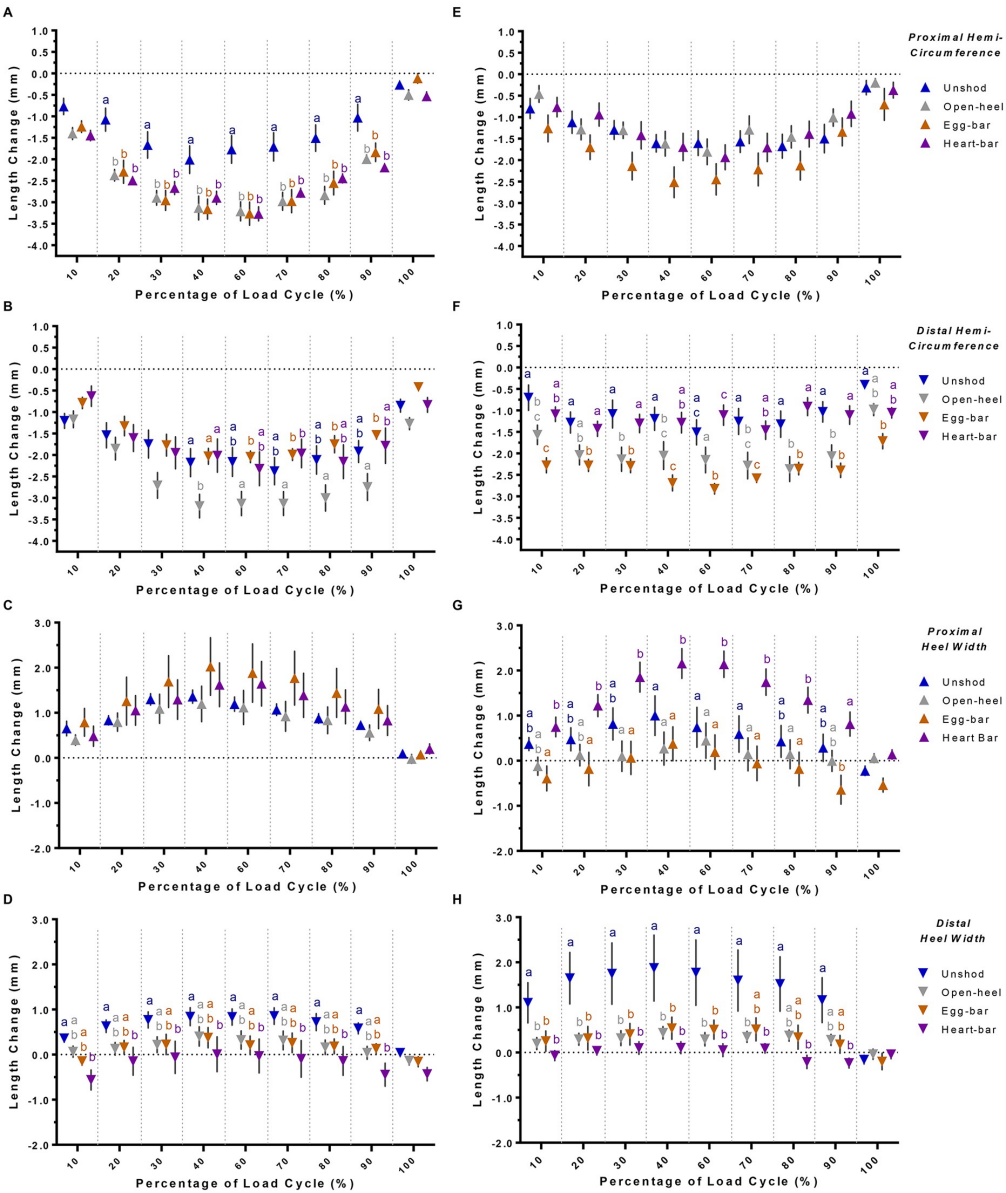
3-D hoof wall deformation. Three-dimensional change (mean ± SEM) of the proximal (A & E) and distal (B & F) hemi-circumference, and proximal (C & G) and distal (D & H) lateromedial heel width in unaffected (A-D) and laminitic (E-H) hooves while unshod (blue) or shod with open-heel (gray), egg-bar (orange), or heart-bar (purple) shoes during a compressive load cycle (1.0x10^2^–5.5x10^3^ N). Significant differences among shoeing types within hoof at specific points in the load cycle are indicated by distinct letters (p≤0.05).

Three-dimensional proximal and distal hemi-circumferences decreased with increasing load. In general, the proximal and distal hemi-circumferences tended to decrease less in laminitic versus unaffected hooves (Fig 8), but there were clear distinctions among shoes within hoof condition. Specifically, the proximal hemi-circumference changed least when unshod versus all shoes in unaffected hooves across loading and unloading phases. However, in laminitic hooves, the decrease in proximal hemi-circumference was similar while unshod and with all shoes. The decrease in distal hemi-circumference was higher in unaffected hooves when shod with open-heel shoes compared to unshod or when shod with egg-bar and heart-bar shoes. The decrease in distal hemi-circumference of laminitic hooves was similar when shod with open-heel or egg-bar shoes, and both were greater than when unshod or shod with heart-bar shoes.

In general, increases in distal hoof heel width were lower with shoes than while unshod in both unaffected and laminitic hooves, and changes in distal heel width were lowest in unaffected and laminitic hooves with heart-bar shoes (Fig 8). Specifically, increases in distal heel width were significantly lower in unaffected hooves with heart-bar shoes relative to unshod and in laminitic hooves with heart-bar shoes versus open-heel, egg-bar, and heart-bar shoes. Increases in proximal heel width were significantly higher in laminitic hooves with heart-bar shoes versus all other shoeing conditions, and greatest in unaffected hooves with open-heel and heart-bar shoes, though not significantly. Changes in proximal heel width and proximal hemi-circumference were inversely related with and without shoes. Additionally, changes in proximal and distal heel width tended to be inversely related for heart-bar and egg-bar shoes in unaffected hooves and heart-bar shoes in laminitic hooves; a lower distal heel width change corresponded to a greater proximal heel width change. Overall, as the proximal hemi-circumference decreased, the proximal heel width increased, and, for some shoes, minimal increases in distal heel width corresponded to greater increases in proximal heel width.

**Fig 9:**
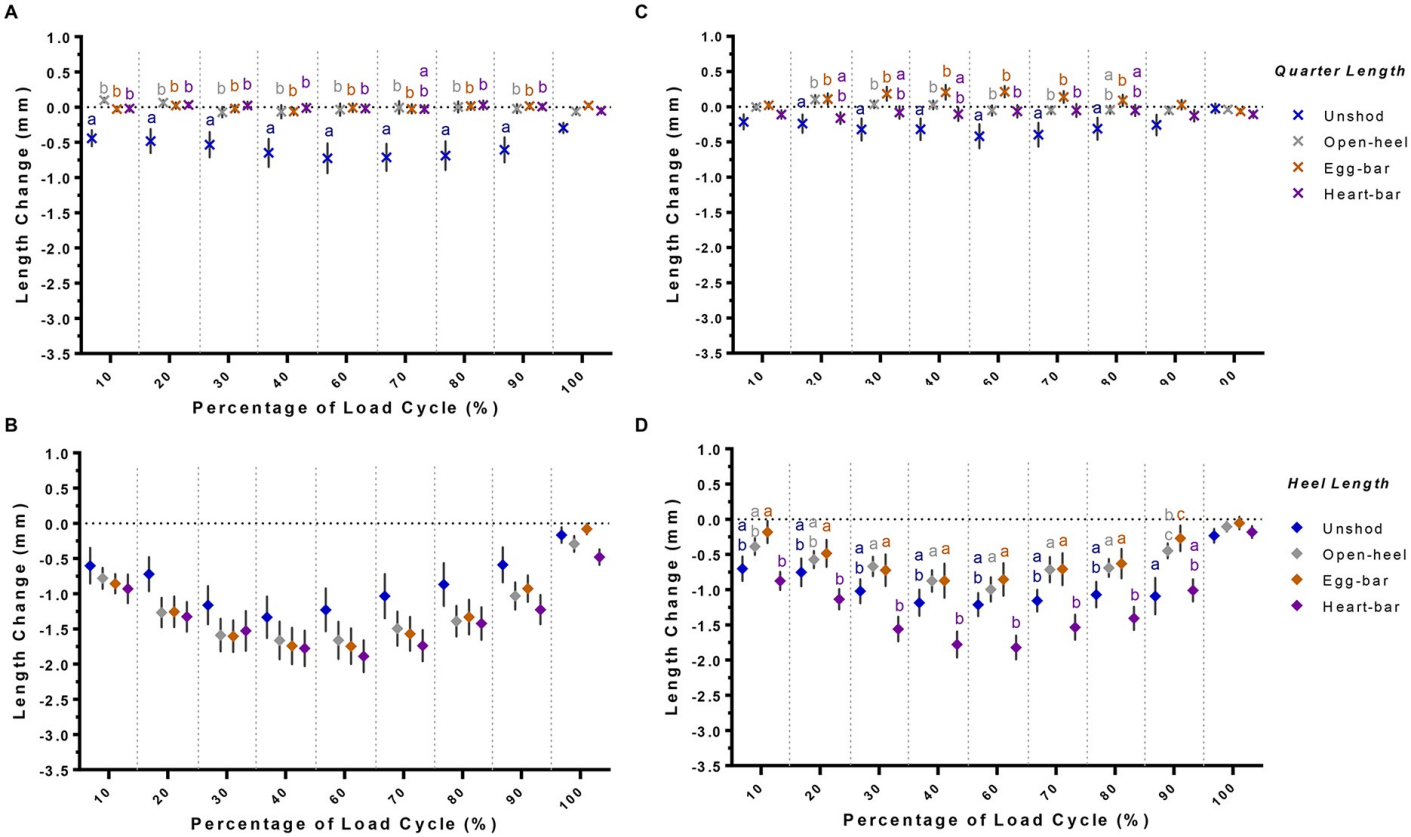
3-D quarter and heel length change. Three-dimensional change (mean ± SEM) of the quarter (A & C) and heel (B & D) length in unaffected (A-B) and laminitic (C-D) hooves while unshod (blue) or shod with open-heel (gray), egg-bar (orange), or heart-bar (purple) shoes during a compressive load cycle (1.0x10^2^–5.5x10^3^ N). Significant differences among shoeing types within hoof at specific points in the load cycle are indicated by distinct letters (p≤0.05).

All three shoe types tended to limit decreases 3-D in quarter length observed in unshod unaffected and laminitic hooves (Fig 9). The heel length decreased similarly while unshod and when shod with all three shoe types in unaffected hooves. Laminitic hooves shod with heart-bar shoes had a greater decrease in heel length during load application than while shod with egg-bar or open-heel shoes.

### Radiographic hoof imaging during loading

**Fig 10:**
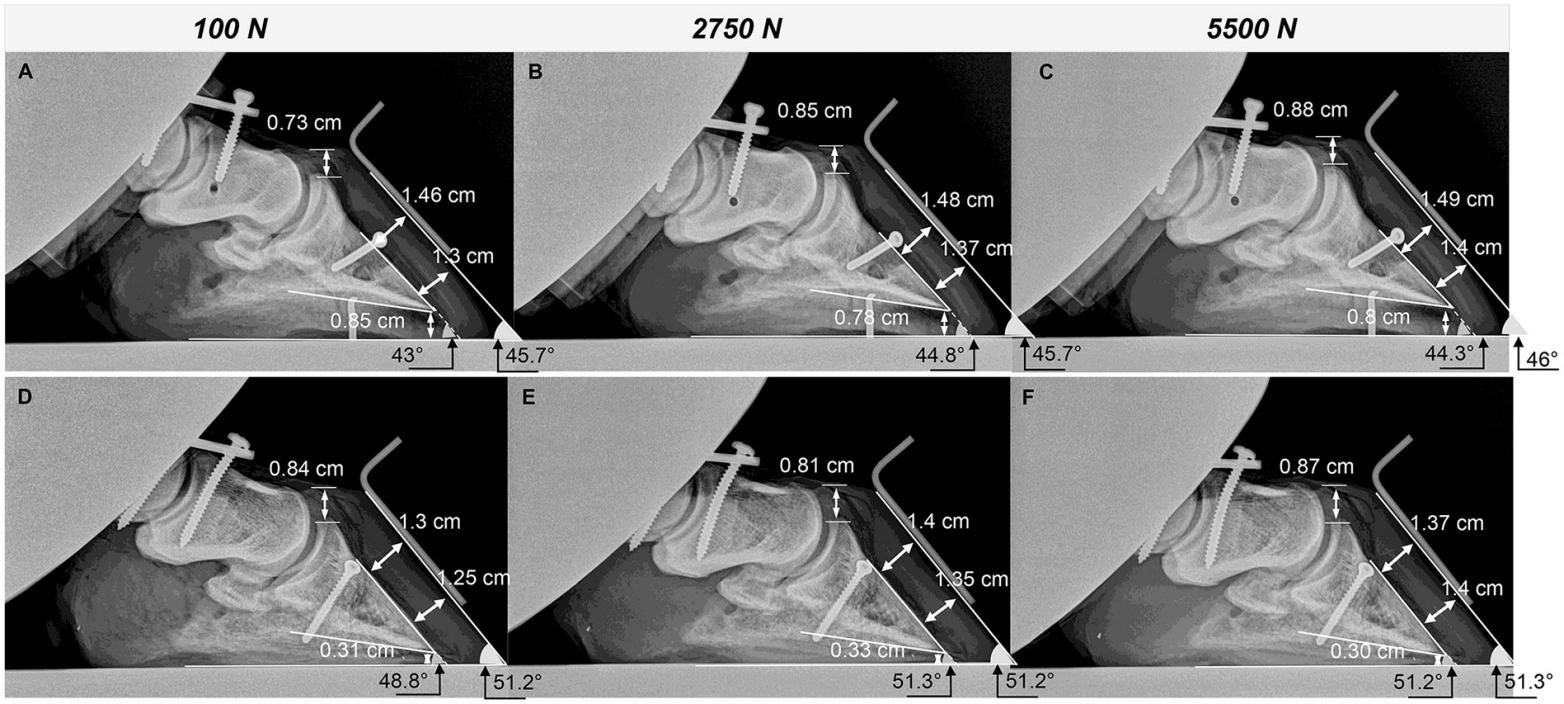
Mediolateral radiographs of unaffected and laminitic hooves with representative measures. Radiographs of an unaffected (upper) and laminitic (lower) equine hoof during compressive loading at 1x10^2^ (A, D), 2.75x10^3^ (B, E), and 5.5x10^3^ N (C, F). Representative measures are indicated on each image.

For purposes of this study, descriptive radiographic measures (Table 1) are provided for unshod specimens only, of which a sample is illustrated in Fig 10. The CE in unaffected hooves changed inconsistently with increasing load and was similar at minimum and maximum loads. The proximal and distal HL generally increased with increasing load in all hooves, but with greater magnitude in the laminitic cohort. Specifically, in laminitic hooves, HL began increasing at low loads to a maximum increase of about 1.2 mm proximally and 1.4 mm distally at the highest load. In unaffected hooves, HL increased more gradually at the proximal and distal third of the P3 parietal surface, about 0.56 mm and 0.72 mm at maximum and minimum load, respectively. The sole depth decreased with compression in unaffected hooves for a total change of about 0.9 mm at full load. However, in laminitic hooves the sole depth decreased about 0.3 mm at 25% of the full force (1375 N) in laminitic hooves, then gradually returned to baseline with increasing load. The dorsal wall angle tended to be higher in unaffected versus laminitic hooves and changed slightly with increasing load while the increase in the P3 parietal surface angle in unaffected hooves was about twice that in laminitic hooves, 2.4° and 1.2°, respectively. Notably, laminitic hooves had a higher CE and proximal and distal HL, when compared to unaffected hooves especially at 75% and 100% of the highest load (4750 and 5500 N, respectively).

**Table 1:** Two dimensional radiographic measures (mean ± SEM) in unaffected and laminitic hooves during loading from 1.0x10^2^ to 5.5x10^3^ N. Measurements included the coronary-extensor distance (CE), the horn lamellar zone width at the proximal (P_HL) and distal (D_HL) third of the P3 parietal surface, the sole depth (S), and the dorsal hoof wall and P3 parietal surface angles.

*Measure*	CE (mm)	P_HL (mm)	D_HL (mm)	S (mm)	Dorsal Wall Angle (°)	P3 Parietal Surface Angle (°)
*Load (N)*						
**100 (U)**	8.02 ± 0.59	16.56 ± 0.84	15.07 ± 1.08	7.23 ± 1.30	49.92 ± 1.85	43.90 ± 0.78
**100 (L)**	9.97 ± 0.53	16.92 ± 1.21	17.33 ± 1.33	5.30 ± 1.53	47.53 ± 1.14	47.90 ± 1.38
**1375 (U)**	8.42 ±0.82	16.72 ± 0.70	15.15 ± 1.07	6.46 ± 1.04	50.49 ± 2.15	45.49 ± 0.88
**1375 (L)**	9.63 ± 0.57	17.69 ± 1.39	18.40 ± 1.73	5.00 ± 1.27	47.78 ± 1.22	49.35 ± 1.07
**2750 (U)**	8.08 ± 0.58	17.01 ± 0.81	15.55 ± 1.29	6.69 ± 1.30	50.58 ± 1.63	46.42 ± 0.73
**2750 (L)**	9.55 ± 0.62	17.72 ± 1.35	18.28 ± 1.56	5.08 ± 1.24	47.66 ± 1.32	50.21 ± 1.16
**4125 (U)**	8.31 ± 0.66	16.95 ± 0.85	15.51 ± 1.09	6.11 ± 0.91	50.16 ± 1.91	46.60 ± 1.60
**4125 (L)**	10.16 ± 0.78	18.09 ± 1.46	18.39 ± 1.48	5.09 ± 2.35	47.97 ± 0.87	48.82 ± 1.19
**5500 (U)**	8.33 ± 0.70	17.12 ± 0.84	15.79 ± 1.55	6.35 ± 1.01	50.11 ± 1.79	46.31 ± 1.59
**5500 (L)**	9.96 ± 0.96	18.12 ± 1.58	18.77 ± 1.69	5.33 ± 1.94	47.87 ± 0.99	49.10 ± 1.13

## Discussion

The results of this in vitro study substantiate distinct effects of shoe configuration on P3 displacement and hoof wall deformation that were dependent on hoof condition, unaffected or laminitic. The first hypothesis, motion of P3 and hoof wall deformation are greater in laminitic versus unaffected hooves regardless of shoe type, was rejected because P3 displacement was only greater in laminitic hooves when unshod or shod with open-heel shoes, and both egg-bar and heart-bar shoes reduced P3 motion in laminitic hooves to that in unaffected hooves shod similarly. Additionally, hoof wall deformation in laminitic hooves tended to be similar to unaffected hooves. The second hypothesis, P3 displacement and hoof wall deformation are greatest while unshod, less with open-heel, then egg-bar shoes, and least with heart-bar shoes applied in laminitic and unaffected hooves, was also rejected since hoof wall deformation was distinct among shoes but did not follow the anticipated order. Furthermore, P3 displacement was similar among shoes in unaffected hooves and, in laminitic hooves, it was greatest with open-heel, then unshod, and least with egg-bar shoes and heart-bar shoes, which had comparable P3 displacement. Egg-bar and heart-bar shoes increased P3 displacement away from DW in unaffected hooves relative to unshod while they decreased it in laminitic hooves, and both open-heel and egg-bar shoes tended to increase P3 motion away from the CB in laminitic hooves while heart-bar shoes tended to decrease P3 motion toward SM the most among shoes in both unaffected and laminitic hooves. Overall, study results suggest that, among the shoes tested, heart-bar shoes provide the greatest P3 stability in laminitic hooves and prevent P3 displacement away from the dorsal hoof wall. They also highlight that shoe configuration alters regional hoof deformation from the unshod state and that the effect varies between unaffected and laminitic hooves. Taken together, the findings of this study provide unique data about P3 motion and hoof deformation in laminitic and unaffected hooves to help guide shoe selection and design for individual hoof condition.

Measures on radiographic images of unshod hooves during application of axial load confirmed P3 rotation and sinking in laminitic hooves at a standing load (30% of full weight, ~1375 N) that was consistent with established diagnostic parameters, despite some overlap in measures between hoof conditions. Specifically, the mean HL, CE, and dorsal wall angle of normal hooves were within the normal ranges of 14–22 mm, 2–15 mm, and 48–54°, respectively, and the distal HL width was lower than the proximal [43, 44]. In contrast, the distal HL was higher than the proximal in laminitic hooves, and both were greater than 17 mm [45]. The sole depth of the laminitic hooves was also lower than that in unaffected hooves, consistent with clinical observations, and the range in sole depth in unaffected hooves, 1.12 mm, versus that in unaffected hooves, 0.33 mm, likely a result of less compressible tissue. Though measures did not consistently change with incremental loading, the mean values of the radiographic measures at a standing load were consistent with diagnostic criteria for chronic laminitis considering inherent variability among individual horses with chronic laminitis [43–45].

The dynamic displacement of P3 quantified over a continuous loading cycle provides a more comprehensive perspective than two-dimensional radiographic exposures at incremental loads. Three-dimensional displacement outcomes confirm the complexity of hoof tissue mechanics that are undoubtedly accentuated in laminitic hooves with variable lamellar detachment [23, 39, 46]. While tissue mechanics were not specifically evaluated in this study, 3-D P3 displacement illustrated in Fig 5 suggests a greater loss of tissue elasticity versus stiffness. The shape of 3-D P3, CB, SM, and DW displacement curves are relatively similar between laminitic and unaffected unshod hooves during the loading phase of the cycle. However, the curves during unloading have a steeper slope than those for unaffected hooves. The same phenomenon is present in curves in hooves with open-heel shoes. Notably, application of egg-bar and heart-shoes appeared to largely restore the 3-D displacement curve shape to that of unaffected hooves. Diminished tissue elasticity is also evident in the incremental increase in the distance between P3 and DW in unshod laminitic hooves that did not decrease with unloading in Fig 7. Based on these results, egg-bar and heart-bar shoes appeared to provide sufficient stability to hoof tissues to maintain 3-D P3 and hoof surface motion similar to that of unaffected hooves. However, the stabilizing effects appear to be most prominent during unloading versus loading during the stance phase.

The finding that P3 motion varies between unloading and loading in laminitic hooves also highlights the importance of responses to shoe configurations that are distinct from unaffected hooves in which the majority of work has been done [28, 29, 31–33, 47–51]. The stabilizing effects of egg-bar and heart-bar shoes on 3-D P3 motion in laminitic hooves were anticipated since the shoes are intentionally designed to shift the center of pressure to the palmar hoof structures during weight bearing to decrease the moment arm around the distal interphalangeal joint and deep digital flexor tendon tension on P3 [22, 23, 31, 36, 38, 52–55]. However, egg-bar and heart-bar shoes had opposite actions between hoof conditions; the shoes increased P3 displacement away from DW relative to unshod in normal hooves and reduced abnormal P3 displacement away from DW relative to unshod in laminitic hooves. All three shoes tended to decrease P3 displacement away from DW in laminitic hooves when compared to unshod, consistent with reducing P3 rotation, though the increased P3 displacement away from DW in the affected hooves might be a source of tissue trauma.

The distance between P3 and CB increased over the unshod condition in laminitic hooves with open-heel and egg-bar shoes to a greater magnitude than in unaffected hooves, so it is possible the shoes promote P3 sinking in weakened hooves. Displacement of P3 toward SM had a similar pattern over the loading cycle for the same shoes in both laminitic and unaffected hooves, though the distance tended to decrease more compared to unshod in unaffected hooves, potentially due to a thicker sole than the laminitic hooves. Both open-heel and egg-bar shoes reduced displacement toward SM compared to unshod in unaffected hooves, but hooves with heart-bar shoes had the lowest P3 displacement toward SM. Significant differences in displacement from unshod and other shoes in unaffected hooves with heart-bar shoes were evident across the loading cycle, but they were most apparent in the unloading phase of laminitic hooves with heart-bar shoes, consistent with the findings above and suggesting a loss of lamellar elasticity [16, 23]. Furthermore, a lower P3 displacement achieved with shoeing, may possibly help limiting the lamellae stretching thus leading to a more comfortable patient [56]. These results confirm important differences among shoe configurations on P3 displacement within the hoof capsule between unaffected and laminitic hooves indicating that, for the best translational value, shoe effects should be evaluated for specific tissue conditions.

Subtle differences in material properties among regions of the hoof result in variable responses to tensile [57–59] and compressive stresses [57, 60]. Similar to shoe effects on P3 displacement, hoof capsule deformation also differed between laminitic and unaffected hooves. The equine hoof is a tough structure with the capacity to absorb high energy through a combined stiff outer stratum medium attached via a ductile soft tissue interface to a bony P3 which is an integral part of the suspensory apparatus through its attachment to the deep digital flexor tendon [58, 59, 61–64]. Diminished mechanical properties of the stratum internum in the laminitic hoof likely impact the dynamic relationship between adjacent hoof components, the external stratum medium, and innermost P3. This is evident in the differences in effects of shoe configuration on proximal and distal hemi-circumferences measured in this investigation. The sum of the hemi-circumference segment distance in 3-D space is not equivalent to a 2-D perimeter of the wall surface; the measures represent changes in relative marker positions in space over the course of a load cycle compared to the original position and are intended for comparative purposes only. In unaffected hooves, the proximal hemi-circumference changed similarly with all shoes resulting in an overall negative change in the sum of 3-D distance between adjacent markers. Only the open-heel shoe seemed to have a similar effect on the distal hemi-circumference. In contrast, in laminitic hooves, shoes did not have a large effect on the proximal hemi-circumference, but the distal hemi-circumference changed negatively with open-heel and egg-bar shoes. This seems to indicate that the heart-bar shoes provide the greatest protection to shape change in the distal aspect of laminitic hooves, confirming the value of the shoe to protecting the weakest damaged tissue, though it was not significantly different from unshod.

The effect of shoe configuration on hoof wall deformation varies between unaffected and laminitic hooves. Differences in shoeing effects on changes in proximal and distal hemi-circumferences between unaffected and laminitic hooves is likely related to compromised tissue connectivity in the latter, as indicated above. The lower increase in distal hemi-circumference in unaffected hooves with egg-bar and heart-bar shoes is consistent with shifting the force concentration to the palmar hoof. Shoes tested in this study seem to concentrate forces on the proximal unaffected hoof with only open-heel shoes having a similar effect on the proximal and distal hemi-circumferences, while egg-bar and heart-bar shoes appearing to create a differential effect with lower deformation of the distal hemi-circumference. An important finding in this investigation is the inverse relationship between proximal hemi-circumference and proximal heel width in both unaffected and laminitic hooves with shoes that are distinct from the unshod condition. With shoes, decreases in proximal hemi-circumference corresponded to increases in proximal heel width. Without shoes, both proximal and distal heel widths increased with decreased proximal hemi-circumference, more so in laminitic hooves. On the other hand, all shoes minimized changes of the quarter lengths when compared to unshod in both unaffected and laminitic hooves. The findings above are further supported by a greater decrease in heel length in laminitic hooves with heart-bar shoes compared to other shoeing conditions. Overall, it appears that shoes may alter normal energy dissipation from deformation of the distal heel, and the function shifts to the proximal heel. The effect is most evident in laminitic hooves with heart-bar shoes consistent with greater tissue stabilization. The consequences of altering heel expansion and concentrating it on the proximal aspect may be an important avenue for future study.

The results of this study are limited by the in vitro nature of the study itself, sample preservation, and sample numbers that represent a small, heterogeneous study population. The load cycle had a higher limit than what might be expected physiologically, and, while 5 replicates of all conditions were obtained, they represent only a single observation period [15–18]. An important point relevant to the findings related to hoof stiffness and elasticity is that the rate of loading and unloading and stance duration was much lower than that of a horse step cycle. While the slower rate allowed repeatable and reliable data collection within the limits of the data collection system, it also resulted in tissue behavior that was distinct from in vivo conditions. Specifically, tissue stiffness and elasticity were likely considerably lower than in viable tissue over a step cycle [65, 66]. Thus, while the mechanical loading cycle utilized in this study supported comparisons among distinct shoes in unaffected and laminitic hooves, the testing conditions do not represent in vivo conditions. Further, the hoof is connected to the highly complex suspensory apparatus via the insertion of the DDFT on P3. Use of only the distal limb in this study eliminated the vast majority of the multiple structures that comprise the suspensory apparatus. The lack of the suspensory apparatus and proximal limb as well as immobilization of the proximal interphalangeal joint may have contributed to higher than physiologic stresses on P3. Despite the inclusion criteria, hoof condition likely varied, especially among horses with chronic laminitis, and it, no doubt, increased variability among samples. Data reduction was intentionally designed to eliminate sample bias by normalizing displacements to the starting position within tests. Though every effort was made to maintain the structural integrity of the hoof, including reusing nail holes to limit the number in the hoof wall, some structural damage that may have influenced structural properties was inevitable. The effect of hoof shape and size as well as animal characteristics like age are considerations for future studies that were not considered here given the small sample sizes. Within the boundaries of the acknowledged limitations above, study results provide previously unavailable knowledge about stabilizing effects of the tested horseshoes on P3 and wall in equine hooves with and without chronic laminitis.

## Conclusion

Study results indicated that out of three tested shoes, open-heel, egg-bar, and heart-bar, only egg-bar and heart-bar configurations decreased P3 motion. Heart-bar shoes had the least P3 sinking in unaffected hooves and less P3 sinking during the unloading phase in laminitic hooves compared to other shoes. In laminitic hooves, heart-bar shoes reduced P3 rotation from the dorsal wall compared to unshod. Open-heel and egg-bar had similar effects on the direction of P3 movement relative to surrounding hoof components. Notably, decreases in proximal hemi-circumference with shoes applied corresponded to increases in proximal heel width in contrast to increases in both proximal and distal heel widths with decreased proximal hemi-circumference in unshod hooves. As such, restricted heel expansion with shoes may alter normal energy dissipation from distal heel deformation and shift it to the proximal heel. In general, shoes reduced hoof quarter compression. Outcomes highlight the differential effects of shoe configuration between clinically normal and laminitic hooves. The findings include dynamic effects of shoe configuration on P3 displacement and hoof deformation during load application to guide shoe selection and design for optimal hoof protection and performance.

## Supporting information

S1 Data (ZIP)
